# Behavioral and Neural Activity-Dependent Recanalization of Plugged Capillaries in the Brain of Adult and Aged Mice

**DOI:** 10.3389/fncel.2022.876746

**Published:** 2022-05-26

**Authors:** Patrick Reeson, Ben Schager, Myrthe Van Sprengel, Craig E. Brown

**Affiliations:** ^1^Division of Medical Sciences, University of Victoria, Victoria, BC, Canada; ^2^Department of Psychiatry, University of British Columbia, Vancouver, BC, Canada

**Keywords:** capillary, cerebral blood flow, neurovascular coupling, GABA, vascular dementia, environmental enrichment, capillary stalling

## Abstract

The capillaries of the brain, owing to their small diameter and low perfusion pressure, are vulnerable to interruptions in blood flow. These tiny occlusions can have outsized consequences on angioarchitecture and brain function; especially when exacerbated by disease states or accumulate with aging. A distinctive feature of the brain’s microvasculature is the ability for active neurons to recruit local blood flow. The coupling of neural activity to blood flow could play an important role in recanalizing obstructed capillaries. To investigate this idea, we experimentally induced capillary obstructions in mice by injecting fluorescent microspheres and then manipulated neural activity levels though behavioral or pharmacologic approaches. We show that engaging adult and aged mice with 12 h exposure to an enriched environment (group housing, novel objects, exercise wheels) was sufficient to significantly reduce the density of obstructed capillaries throughout the forebrain. In order to more directly manipulate neural activity, we pharmacologically suppressed or increased neuronal activity in the somatosensory cortex. When we suppressed cortical activity, recanalization was impaired given the density of obstructed capillaries was significantly increased. Conversely, increasing cortical activity improved capillary recanalization. Since systemic cardiovascular factors (changes in heart rate, blood pressure) could explain these effects on recanalization, we demonstrate that unilateral manipulations of neural activity through whisker trimming or injection of muscimol, still had significant and hemisphere specific effects on recanalization, even in mice exposed to enrichment where cardiovascular effects would be evident in both hemispheres. In summary, our studies reveal that neural activity bi-directionally regulates the recanalization of obstructed capillaries. Further, we show that stimulating brain activity through behavioral engagement (i.e., environmental enrichment) can promote vascular health throughout the lifespan.

## Introduction

There is growing appreciation of the unique vulnerabilities of the cerebral blood supply. Specifically the capillary bed, which comprises >90% of vascular length and primarily distributes blood flow throughout the brain, is prone to spontaneous obstructions by cells or circulating debris (Blinder et al., 2013; Santisakultarm et al., 2014; Gould et al., 2017; Reeson et al., 2018; Erdener and Dalkara, 2019; Bracko et al., 2020; Schager and Brown, 2020). These obstructions can be ephemeral or persistent, and are far more abundant in disease states (Santisakultarm et al., 2014; Cruz Hernández et al., 2019; Bracko et al., 2020; Ali et al., 2022). Given the fact that persistently obstructed capillaries are eventually pruned (Reeson et al., 2018), any intervention that promotes capillary recanalization could conceivably preserve blood supply and buttress the vasculature’s ability to support cognitive functions. It has been demonstrated in a variety of species, including humans, that as we age the density of capillaries in the brain decreases (Buchweitz-Milton and Weiss, 1987; Riddle et al., 2003; Brown and Thore, 2011; Harb et al., 2013; van Dinther et al., 2022). This age-related loss of vessel density correlates with impaired cognitive function, and can be partially predicted by brain region specific vulnerability to capillary plugging (Mann et al., 1986; Iadecola, 2013; Langdon et al., 2018; Schager and Brown, 2020; Yoon et al., 2022). Rodent studies have demonstrated that depleting adherent neutrophils can improve cerebral blood flow and performance on sensory-motor and cognitive tasks in mouse models of Alzheimer’s disease and stroke (Cruz Hernández et al., 2019; El Amki et al., 2020). While concerted efforts across multiple labs (Reeson et al., 2018; Cruz Hernández et al., 2019; Erdener et al., 2019; Yoon et al., 2022) have begun to understand the phenomenology of capillary obstructions, there remain serious gaps in our understanding of the mechanisms that regulate capillary recanalization.

The majority of obstructed cortical capillaries that recanalize, do so by extruding the obstruction back into the circulation (Reeson et al., 2018). This suggests that changes in vascular tone and blood flow, could be effective in enhancing recanalization after obstruction. The dynamic regulation of cerebral blood flow to meet the metabolic demands of active neurons is referred to as neuro-vascular coupling (NVC) (Gordon et al., 2007; Attwell et al., 2010; Hall et al., 2014; Hill et al., 2015; Ma et al., 2016; Mishra et al., 2016). From a mechanistic perspective, increased neural activity and resultant metabolic demands to restore ionic gradients (Attwell and Laughlin, 2001), trigger the recruitment of local blood flow through intermediate and effector cells such as astrocytes, mural cells, and endothelium (Devor et al., 2003; Gordon et al., 2007; Kleinfeld et al., 2011; Hall et al., 2014; Mishra et al., 2016; Lecrux et al., 2019; Grubb et al., 2020; Hartmann et al., 2021). However, the activity of cortical neurons is powerfully regulated by local inhibitory interneurons that release GABA (Uhlirova et al., 2016; Echagarruga et al., 2020). Therefore, it stands to reason that modulating neural activity levels, either through pharmacological or behavioral interventions such environment enrichment, could bi-directionally regulate the recanalization of obstructed capillaries.

A major challenge that limits the mechanistic study of capillary recanalization, is the relatively low prevalence of long lasting obstructions (Santisakultarm et al., 2014; Reeson et al., 2018; Erdener et al., 2019), and the inherent difficulties in detecting/visualizing obstructions. One approach to circumvent these issues has been to induce capillary obstructions through intravenous injection of fluorescent microspheres (Reeson et al., 2018; Schager and Brown, 2020). While not necessarily “naturalistic,” this approach allows one to easily identify capillaries that are prone to stalling. Critically this approach enables the experimenter to control the timing of obstructions, and thus isolate different epochs of recanalization. Previously we have shown that fluorescent microspheres not only recapitulate key characteristics of natural obstructions, they also do not lead to micro-infarcts or systematic cardiovascular changes which could confound results (Reeson et al., 2018). Here we used experimentally induced obstructions to test whether simply enriching a mouse’s home cage for just 12 h could facilitate recanalization. We further disambiguated systematic cardiovascular effects from local neuronal activation through targeted sensory deprivation and pharmacological approaches. Our experiments reveal that neural activity bi-directionally regulates the recanalization of obstructed capillaries in adult and aged mice.

## Materials and Methods

### Animals

Experiments involved male and female adult and aged mice (3–4 vs. 16–19 months old) on a C57/BL6J or FVB/NJ background (Jax Strain #003658). Experimental cohorts consisted of littermates matched for age and sex and when possible. Due to limited availability and therefore sampling in aged mice, we did not explicitly test for an effect of sex and therefore data was pooled across sex. All mice were housed under a 12 h light/dark cycle with *ad libitum* access to water and standard laboratory diet. All experiments were conducted according to the guidelines set by the Canadian Council of Animal Care and approved by the University of Victoria Animal Care Committee.

### Experimental Treatments

**FIGURE 1:**
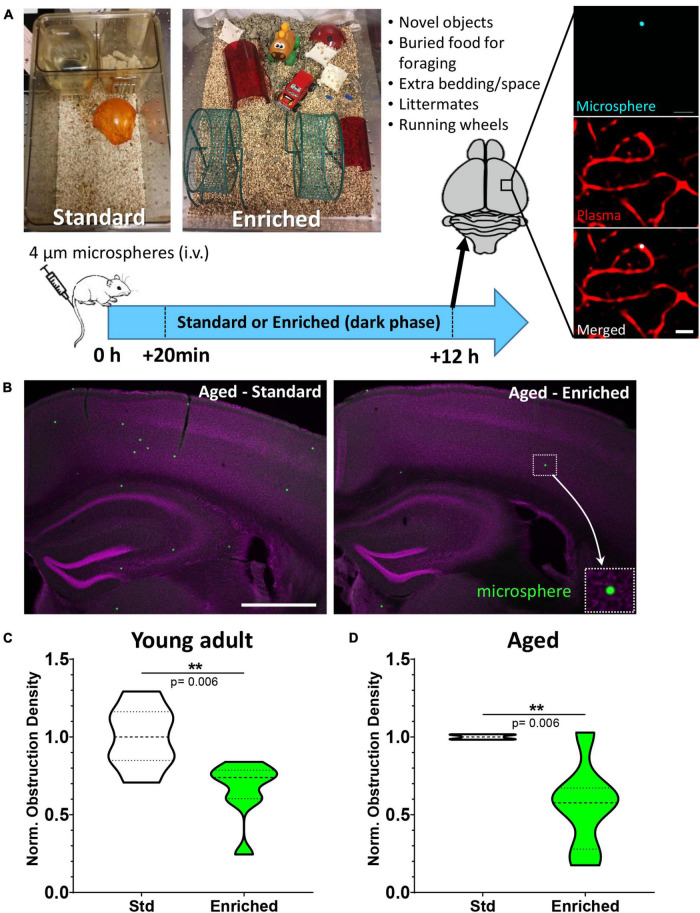
Environmental enrichment enhances capillary recanalization in the cortex of young adult and aged mice. **(A)** Images show standard and enriched cages and timeline of experiments. Far right panel shows *ex vivo* confocal images of a microsphere lodged within the lumen of a capillary. **(B)** Representative images of microspheres distributed across cortical and subcortical regions 12 h after injection in aged mice exposed to standard or enriched environment (microspheres pseudocolored in green, magenta represents DAPI labeling). **(C)** Normalized cortical microsphere density for young adult mice housed in standard or enriched cages [*n* = 7 and 8 mice, respectively; unpaired *t* test *t*_(13)_ = 3.31, *p* = 0.006]. **(D)** Microsphere obstruction density in 16–19 month old male (8) and female (4) mice placed in either standard (*n* = 5) or enriched (*n* = 7) cage for 12 h. Data was normalized to the mean cortical obstruction density of standard housed mice for each independent cohort. Aged mice exposed to enrichment had a significant reduction in cortical obstructions [0.56 ± 0.28, *t*_(10)_ = 3.51 *p* = 0.006]. **p < 0.01. Scale bars = 20 μm **(A)** or 1 mm **(B)**.

For blood plasma labeling, lysine fixable FITC dextran or Texas Red (100 μL; 2% w/v in 0.9% saline; ThermoFisher, molecular weight 40 kDa, D1845) was intravenously injected into isoflurane anesthetized mice (1.5% mixed in medical air) and allowed to circulate for 5–8 min prior to decapitation. For inducing capillary obstructions, 20 μL of fluorescent microspheres (4 μm diameter; 2% solids; Life Technologies FluoSpheres sulfate, F8858) were mixed with 100 μL of saline and injected into the tail vein or retro-orbitally in aged mice due to it’s higher success rate. A master solution of microspheres was first made, sonicated in Elmasonic S10H (3 min) and then aliquoted into separate 20 μL injection doses which were then assigned to mice randomly. To minimize variability between and within groups, control and experimental mice were run in parallel and received injections from the same stock of microspheres. Twenty minutes after microsphere injections, mice were assigned to one of 3 sets of experiments to test the effect of: (a) environmental enrichment, (b) pharmacological modulation, or (c) whisker trimming on capillary recanalization. We waited 20 min after microspheres injection since there would be very few microspheres still freely circulating (Reeson et al., 2018), given the single pass circulation time in a mouse is ∼15 s, therefore ∼80 cycles of filtration by liver and other organs (Welsher et al., 2011). Enriched cages were approximately three times larger than standard cages (∼600 vs. 1750 cm^2^), housed 2–3 mice, contained extra nesting, hutches, an exercise wheel, novel objects and buried food (see Figure 1A). The standard cage contained a hutch and mice were housed singly. For whisker trimming experiments, all facial whiskers on the left side of the face were trimmed to ∼1–2 mm in length. Lastly, for local manipulation of neural activity, a small hole was drilled through the skull above the right and left forelimb primary somatosensory (FLS1) cortex. In one hemisphere a glass pipette (tip diameter ∼25 μm) was lowered 500 μm below the cortical surface and 0.4 μL of vehicle with lysine fixable FITC dextran (2% w/v in 0.9% saline; ThermoFisher, molecular weight 70 kDa) was pressure injected. The contralateral hemisphere was then similarly injected with either vehicle, GABA_A_ agonist muscimol (50 mM) or inverse agonist L-655,708 (100 μM) dissolved in lysine fixable Texas Red dextran (2% w/v in 0.9% saline; ThermoFisher, molecular weight 70 kDa). The burr holes were sealed with bone wax (F.S.T. 19009-00) and the scalp sutured. Mice quickly recovered under a heat lamp and were housed in standard or enriched cages until euthanasia.

### Tissue Processing, Imaging, and Analysis

Twelve hours after microsphere injections, brains were extracted from deeply anesthetized mice and fixed in 4% paraformaldehyde (PFA) in 0.1M phosphate buffered saline (PBS) overnight at 4°C. Brains were sectioned in the coronal plane at 100 μm thick using a Leica vibratome (T1000). Every third section was mounted on a gelatin-coated slide, and coverslipped with Fluoromount G (ThermoFisher, 00-4958-02). Fluorescently filled vessels with microsphere obstructions were imaged using an Olympus confocal microscope with a 10× objective (NA 0.40). Confocal image stacks were collected in 4 μm z-steps at a pixel resolution of 1.242 μm/pixel. In order to quantify the density of microsphere obstructions across the forebrain, coronal brain sections were imaged on a widefield Olympus BX51 microscope with a 4× UPlanFLN objective (NA = 0.13, 0.72 μm/pixel, 1.15 × 0.87 mm) and an Olympus DP73 digital camera using CellSens software. Images were taken of every 3rd section, sampled from +1.70 to -2.70 mm relative to bregma (Franklin, 2008). Using ImageJ software (Schindelin et al., 2012), an experimenter blinded to condition counted the number of microspheres within cortical regions of interest (e.g., S1BF cortex or dextran labeled cortex) to estimate microsphere density (microsphere obstructions/mm^3^). Since not all experiments could be run at the same time, we ran cohorts where the density of microsphere obstructions in an experimental group was normalized to the average density of that cohort’s control animals. Normalized ratios were then averaged across cohorts (typically each experiment consisted of 2–4 cohorts).

### Validating Effects of GABA_A_ Agonist and Inverse Agonist on Cortical Activity *in vivo*

Mice were lightly anesthetized with 15% urethane dissolved in water (1.25 g/kg). Once tail pinch reflexes were lost, mice were secured into a surgical plate with body temperature maintained at 37°C. A hole was drilled through the skull above the right forelimb somatosensory cortex (FLS1) so a 1–2 MΩ glass micropipette filled with HEPES-buffered Artificial Cerebral-Spinal Fluid (ACSF) could be inserted into the brain 200–300 μm below the cortical surface. Evoked potentials were amplified (1000×) and filtered between 1 and 1000 Hz with a differential amplifier (A-M Systems). A single 5 ms deflection of the forelimb with a piezoelectric wafer (∼300 μm deflection) was used to evoke cortical field potentials every 10 s and averaged over 45 trials. Cortical responses were collected for up to 60 min after topical application of 50 mM GABA_A_ agonist muscimol or 100 μM of the inverse agonist L-655,708.

### Statistics

All statistical analyses were performed using GraphPad Prism 8 using an alpha value of 0.05. *Post hoc* comparisons were performed using unpaired or paired *t*-tests for within cohort or within animal comparisons. Repeated measures ANOVA was used to test for region-related differences in microsphere clearance rates. Data are presented as median ± quartile and maximum and minimum values (violin or box and whisker plots).

## Results

We first tested if increased behavioral engagement and sensory stimulation (running, nesting, novel objects, littermates, exploration) could affect the recanalization of cortical capillaries. Mice were reared and housed in standard cages up until the start of the experiment and then were assigned to standard or enriched environment (see Figure 1A). Previous work from our lab has shown that intravenous injection of fluorescent microspheres (4 μm diameter) can be used as a high throughput approach to quantitatively assess mechanisms of capillary recanalization in the brain (Figures 1A,B) without impairing cardiovascular function (Reeson et al., 2018). We focused on the 12 h post-injection period during the dark phase of the light cycle, when the majority of capillaries would recanalize (Reeson et al., 2018). A subset of mice were also injected (i.v.) with a fluorescent dextran to confirm obstructions were restricted to capillaries (Figure 1A, right panel). Our analysis in adults (3–4 months old) indicated that the density of cerebral microsphere obstructions in mice given standard housing was 74.7 ± 21.6 obstructions/mm^3^, or a normalized density of 1.0 ± 0.2 (Figure 1C). In comparison, adult mice exposed to enriched cages had a significantly lower average density of obstructions [49.9 ± 24.9 obstructions/mm^3^, normalized 0.66 ± 0.2, ratio paired *t*-test of absolute density *t*_(6)_ = 3.53 *p* = 0.01, *t*-test of normalized densities *t*_(13)_ = 3.31 *p* = 0.006, Figure 1C]. Since obstructions to capillary flow can profoundly alter cognitive or sensory-motor function in neurological diseases associated with aging [e.g., Alzheimer’s disease, ischemia, (Taylor et al., 2016; Cruz Hernández et al., 2019; Faulhaber et al., 2022)], a critical question is whether the benefits of enrichment extend to aged animals. Our analysis revealed that aged mice (16–19 months of age) exposed to an enriched cage had dramatically lower obstruction densities when compared to the age matched mice exposed to standard cages (mean 0.56 ± 0.28; Figures 1B,D). While one aged mouse (out of seven) received no boost in recanalization from enrichment (normalized obstruction density 1.03), all other enriched aged mice showed a significant reduction in obstructions ranging from 0.67 to 0.17 of their control littermates [*t*_(10)_ = 3.51 *p* = 0.006]. These experiments demonstrate that behavioral engagement by way of environmental enrichment, significantly enhanced the recanalization of cerebral capillaries in both young adult and aged mice.

**FIGURE 2:**
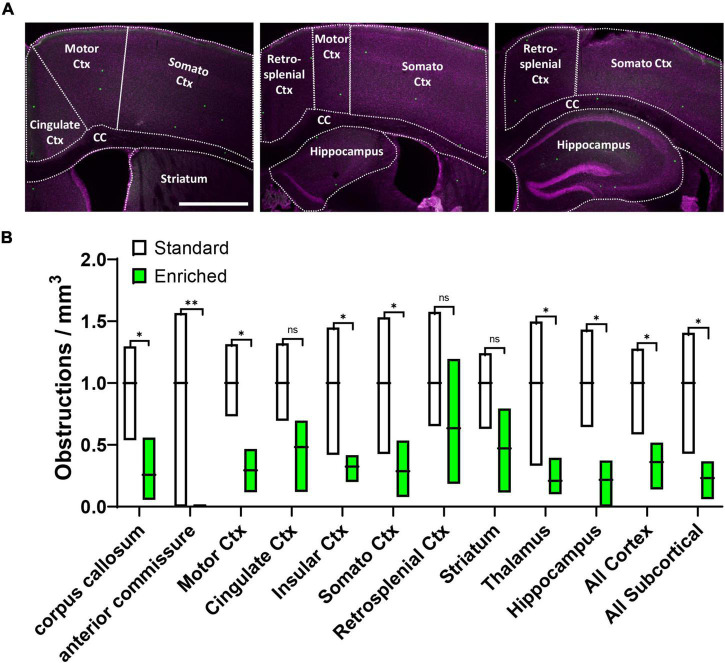
Environmental enrichment generally improves recanalization across different brain regions. **(A)** Confocal images showing different brain regions outlined for analysis. **(B)** Box plot shows the obstruction density across different brain regions in aged mice exposed to standard or enriched cages. Note that striatum, thalamus, and hippocampus comprised the “All Subcortical” group. n.s. = not significant, *p < 0.5, ^**^p < 0.01. Scale bar = 1 mm.

Although our results clearly show that environmental enrichment improves capillary recanalization in the cortex, it was unclear if this benefit would be more evident in specific cortical areas or extend to subcortical regions. Therefore, in aged mice we further measured obstruction densities within specific cortical and subcortical areas aligned to a standard mouse brain atlas, and normalized densities within each region to standard housed controls (Figure 2A). Our analysis revealed that the effects of enrichment on capillary obstruction density were evident across all regions examined [Figure 2B, 2-way ANOVA main effect of Enrichment, *F*_(1_,_48)_ = 54.40, *p* < 0.0001] Although most regions showed a significant reduction in obstruction density with enrichment, a few regions had higher levels of variability and thus did not achieve statistical significance (Cingulate Cortex *p* = 0.11, Retrospenial Cortex *p* = 0.26, and Striatum *p* = 0.10). Collectively, these results suggest a forebrain-wide benefit of enrichment on capillary recanalization.

**FIGURE 3:**
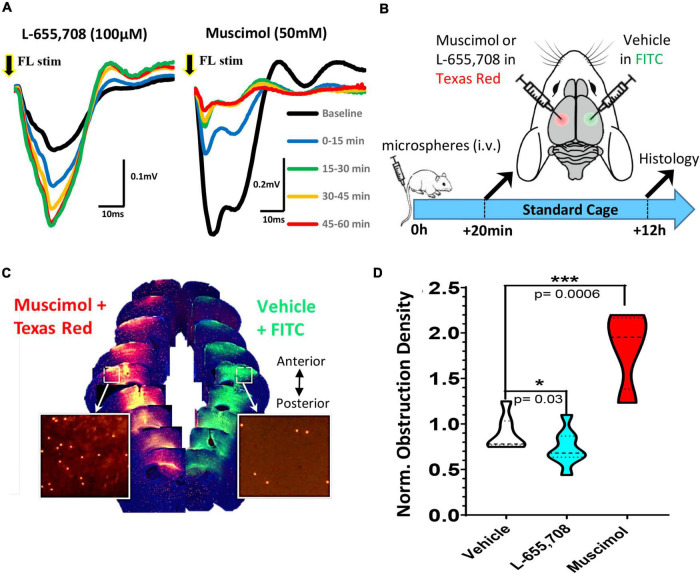
Pharmacological modulation of neuronal excitability reveals bi-directional regulation of cortical capillary recanalization. **(A)** Field potentials were recorded in the right forelimb somatosensory cortex (FLS1) in response to a single 5 ms deflection of the contralateral forelimb either at baseline (black trace) or after topical application of 100 μM L-655,708 (left panel) or 50 mM muscimol (right panel). Each trace represents the average of 90 stimulation trials delivered over 15 min epochs. Note the rapid and sustained increase in response amplitude after L-655,708 or decrease after muscimol. **(B)** Timeline of pharmacology experiments. Twenty minutes after i.v. injection of microspheres, young adult mice received a microinjection of either L-655,708, muscimol or vehicle (with dextran tracer) into the right somatosensory cortex and a vehicle injection in the opposite hemisphere. **(C)** Image montage of coronal brain sections (anterior to posterior) showing the diffusion of fluorescent dextrans at each injection site and microspheres remaining after 12 h (inserts). **(D)** Comparison of normalized cortical obstruction densities (normalized to vehicle/vehicle injection control group) in each treatment group. L-655,708 significantly reduced obstruction density compared to vehicle injected mice [vehicle: 0.90 ± 0.19 vs. L-655,708: 0.66 ± 0.18, unpaired *t* test *t*_(13)_ = 2.52, *p* = 0.03]. Conversely, muscimol significantly increased capillary obstruction density [muscimol: 1.84 ± 0.43; *t*_(9)_ = 5.161, *p* = 0.0006] relative to vehicle control group. Data are mean ± standard deviation. *p < 0.05, ***p < 0.001.

Given the widespread effects of enrichment on recanalization, both changes in neuronal activity and/or the cardiovascular system (such as elevated heart rate) could account for these benefits. In order to more directly test the role of neural activity in capillary recanalization, we employed a pharmacological approach. To decrease or increase cortical activity, we used either the GABA-A agonist muscimol or inverse agonist L-655,708, respectively. We first validated the effect of each drug on cortical activity *in vivo* by recording sensory evoked field potentials in the somatosensory cortex before and 60 min after topical application. As shown in Figure 3A, the inverse agonist L-655,708 (100 μM) rapidly increased the amplitude of sensory evoked cortical responses (relative to baseline denoted with black line) which persisted for at least 1 h after injection. Conversely, muscimol (50 mM) suppressed cortical responses during this time period. To test the effects of these drugs on recanalization, we first injected microspheres (i.v.) and then 20 min later micro-injected muscimol or L-655,708 into the right somatosensory cortex and vehicle in the opposite hemisphere (Figure 3B). Fluorescent dextrans (2% 70 kDa FITC or Texas Red) were included in solutions to estimate drug diffusion and estimate cortical regions with altered neural activity. Mice were housed in standard cages and then 12 h later, brains were extracted and we compared the density of obstructions in the experimental hemisphere relative to the control (vehicle injected) hemisphere (see Figure 3C for representative example). In both hemispheres we restricted our analysis to cortical regions labeled by the diffusion of fluorescent dextran (Figure 3C). For mice injected with vehicle in both hemispheres, the obstruction ratio was 0.90 ± 0.19, indicating no difference between left and right hemispheres [*t*_(6)_ = 1.49 *p* = 0.187; Figure 3D]. Mice injected with L-655,708 had a lower mean obstruction ratio of 0.66 ± 0.18, indicating capillary recanalization was significantly improved by increasing neural activity [*t*_(13)_ = 2.52 *p* = 0.03]. Additionally, the obstruction ratio in mice injected with muscimol was significantly increased [Figure 3D; 1.84 ± 0.43; *t*_(9)_ = 5.161 *p* = 0.0006]. These results show that capillary recanalization is bi-directionally modulated by neural activity levels.

**FIGURE 4:**
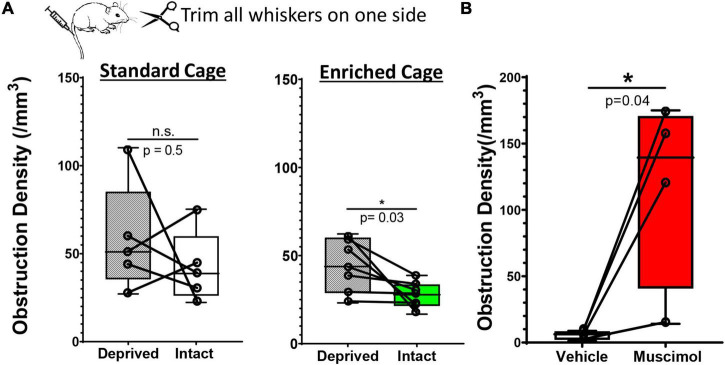
Hemisphere specific changes in neural activity drive recanalization. **(A)** Whisker box plots show the density of microsphere obstructions in the sensory deprived and intact S1BF cortex in young adult mice subjected to unilateral whisker trimming. There was no significant difference between deprived and intact S1BF (somatosensory barrel field) in mice housed in standard cages [intact: 42.17 ± 20.37 obstructions/mm^3^ vs. deprived S1BF: 42.17 ± 20.37 obstructions/mm^3^, paired *t* test, *t*_(4)_ = 0.818, *p* = 0.46, *n* = 5 mice/group]. However, mice exposed to an enriched environment showed a significant reduction in the intact S1BF (27.22 ± 7.63 obstructions/mm^3^) compared to the deprived S1BF [44.42 ± 15.25 obstructions/mm^3^, paired *t* test *t*_(6)_ = 2.66, *p* = 0.03; *n* = 7 mice/group]. **(B)** Plots show the density of microsphere obstructions in vehicle or muscimol injected hemisphere of young adult mice exposed to an enriched environment. Data are mean ± standard deviation. *p < 0.05.

While our findings suggest that enrichment or pharmacological based stimulation of neural activity can improve capillary recanalization, we still could not rule out systematic cardiovascular effects (such as heart rate or blood pressure) as playing a primary role, especially in enriched mice. To address this question, we modulated neural activity in one hemisphere by unilateral trimming all whiskers on the left side of the face. We reasoned that this manipulation would increase or bias sensory driven neural activity in the intact hemisphere relative to the deprived, especially if mice were placed in an enriched environment. Importantly, both cerebral hemispheres in enriched mice would be subjected to systemic cardiovascular effects (increased heart rate or blood pressure) associated with exercise or social engagement. Therefore, two predictions emerge: (i) relative to standard environment, enrichment should enhance the bias in neural activity toward the intact hemisphere, and presumably improve recanalization, (ii) if systemic cardiovascular effects are more important than neural activity, then there should be no differences between intact vs. deprived hemisphere in enriched mice. Our analysis revealed that the obstruction density in the primary barrel-field (S1BF) cortex in mice housed in standard cages was not significantly different between intact versus deprived S1BF cortex [Figure 4A; 42.17 ± 20.3 vs. 58.48 ± 31.4 obstructions/mm^3^, paired *t*-test *t*_(4)_ = 0.082 *p* = 0.5]. For mice exposed to enriched cages, the density of obstructions was significantly reduced in the intact S1BF relative to the deprived region [Figure 4A; 44.42 ± 15.2 vs. 27.22 ± 7.6 obstructions/mm^3^ in Deprived and intact regions, respectively; *t*_(6)_ = 2.66 *p* = 0.03]. These findings argue that the effects of enrichment on recanalization are likely conferred by changes in local neural activity rather than global cardiovascular effects. And finally, given the profound inhibitory effect of muscimol on recanalization, we then asked whether this effect would persist even when systemic cardiovascular parameters are altered during enriched housing (Note: our previous muscimol experiments shown in Figure 3 were conducted using standard cages). As shown in Figure 4B, the effects of muscimol on capillary obstruction density persist even when mice were exposed to an enriched environment [paired *t*-test for vehicle and muscimol treated hemispheres: *t*_(3)_ = 3.23 *p* = 0.04]. In conclusion, if systemic cardiovascular changes were the primary driver of recanalization, we would expect the differences between vehicle and muscimol injected hemispheres to disappear, and clearly, they do not.

## Discussion

In the present study, we asked whether the coupling of blood flow to neural activity could be leveraged to improve capillary recanalization following the intravenous injection of fluorescent microspheres. To non-invasively increase neural activity for 12 h, we used environmental enrichment, which encourages sensory and motor exploration, and has a range of established effects on the rodent brain (He et al., 2017). We focused on the 20 min period after injection (when all circulating microspheres are either lodged or cleared) to 12 h later, which previous research has shown was a critical window for capillaries to recanalize (Reeson et al., 2018). We found that environmental enrichment was alone sufficient to improve recanalization rates across cortical and subcortical regions. Importantly, the effects of enrichment extended to aged mice. We further explored the influence of neural activity on recanalization rates with pharmacological approaches. Either increasing cortical excitability or silencing the cortex was sufficient to drive recanalization rates up or down, respectively. And finally, to disambiguate the role of neural activity versus global cardiovascular effects (such as increased heart rate, blood pressure), we unilaterally augmented cortical activity through whisker trimming and exposed mice to a standard or enriched cage. While the intact and deprived barrel cortex was indistinguishable in standard housed mice, they were significantly different in mice from enriched cages, despite only varying in the degree of sensory driven activity. Collectively, these findings implicate neural activity based changes in capillary blood flow as a primary driver of embolus recanalization in both young adult and aged mice.

To stimulate neural activity, we exposed adult mice to an enriched environment that included novel objects, extra bedding, large cage size, increased social interactions, and an exercise wheel. Environmental enrichment is well known to enhance brain function at any stage of life, facilitate recovery from brain injury or promote resiliency to neurological disease (Kleim et al., 2003; Milgram et al., 2006; Jeffers and Corbett, 2018; Trigiani et al., 2020). With regard to cerebrovascular structure, some studies have shown that stimulating sensory driven activity in early postnatal life or exposing mature mice to environmental enrichment, can increase cerebral microvascular density and branching (Lacoste et al., 2014; He et al., 2017), although opposite effects have been described (Whiteus et al., 2014). Here we extend these findings to show that environmental enrichment has a remarkable impact on capillary recanalization. The idea that components of enrichment, such as exercise, can augment cerebral blood flow and therefore could affect capillary recanalization, is not new. Many rodent studies have shown that exercise can increase capillary blood flow, tissue oxygenation, and microvascular density in the cerebral cortex (Swain et al., 2003; Murugesan et al., 2012; Huang et al., 2013; Moeini et al., 2020). Indirect evidence that exercise might improve recanalization comes from the fact that it could prevent the inevitable consequence of recanalization failure (Reeson et al., 2018), namely, vessel pruning and the appearance of string vessels (Leardini-Tristão et al., 2020; Trigiani et al., 2020). Whether the vascular benefits of exercise are mediated by global cardiovascular changes in heart rate and blood pressure, or by activating sensory afferents of the limbs and engagement of motor systems, is not entirely clear. Our findings suggest it may be mediated through the latter mechanism since hemisphere specific effects on neural activity through whisker trimming or muscimol, were still present in mice subjected to environmental enrichment.

Since systemic cardiovascular factors cannot account for our hemisphere specific effects, one would suspect that neural activity mediated changes in blood flow or neurovascular coupling (NVC), would be the primary mechanism behind this phenomenon. It is well established that increased neuronal spiking leads to local changes in blood flow, capillary tone, and diameter (Gordon et al., 2007; Attwell et al., 2010; Drew et al., 2011; Lecrux et al., 2019). Indeed, we support this hypothesis by showing that direct suppression of neural activity with a GABA_A_ receptor agonist profoundly inhibits recanalization, whereas an inverse agonist that increases cortical excitability stimulates recanalization. Although we did not attempt to directly measure blood flow following drug injections, our results agree with a recent study showing that intracortical muscimol injections significantly decreases basal arteriole diameter, capillary flow velocity, and is capable of blocking locomotion induced vasodilation (Echagarruga et al., 2020). Since vascular endothelial cells and pericytes express little to no transcripts for GABA_A_ receptors (Vanlandewijck et al., 2018), we can be reasonably confident that the bi-directional effects of GABA_A_ receptor modulators were mediated through neural activity dependent changes in blood flow, rather than a direct effect on the vasculature. However, we concede that it is still possible that GABA_A_ receptor modulators could have acted directly on vascular cells (Fergus and Lee, 1997), or perhaps initiated vascular driven changes in neuronal activity (Kim et al., 2016). Future work will be needed to further refine which cortical circuits are involved although recent evidence has implicated interneurons (Anenberg et al., 2015; Uhlirova et al., 2016), particularly those expressing nNOS as key contributors (Lee and Dan, 2012; Echagarruga et al., 2020).

While neural activity dependent changes in blood flow likely underlie the effects described in our study, the fact that enrichment broadly improved recanalization in both cortical and subcortical regions suggest other factors, such as neuromodulatory systems, could be involved. Norepinephrine, serotonin and acetylcholine are released widely throughout the forebrain where they powerfully modulate neuronal excitability (Marder, 2012). These neuromodulatory systems permit prolonged epochs of increased neural activity during various behavioral activities (Lee and Dan, 2012; Thiele and Bellgrove, 2018) and are closely linked to attention, mood and social engagement, all of which would be influenced by enriched housing. Furthermore, norepinephrine and acetylcholine can directly augment sensory driven functional hyperemia (Bekar et al., 2012; Lecrux et al., 2017; Zambach et al., 2021). Thus it is conceivable that our non-invasive approaches for promoting capillary recanalization require a sufficiently strong signal from both sensory inputs and neuromodulatory systems. The role of attention, engagement and neuromodulatory factors could explain why biasing sensory driven neuronal activity with whisker trimming, strongly influenced recanalization when mice were housed in enriched cages, but had no effect under standard conditions. Future studies using pharmacology and chemo/opto-gentic approaches for manipulating neuromodulatory systems, will be needed to fully address this idea.

There are important caveats to be considered in our study. First, we employed a histological approach to infer capillary recanalization rates across multiple brain regions. While this approach has distinct advantages over *in vivo* 2-photon microscopy such as increased sampling across different brain regions (sampling hundreds of microspheres across 10 brain regions vs. 1–3 microspheres in 1 region), as well as letting mice freely explore in their enriched home cages between the start and finish of the 12 h experiment, it does not directly show recanalization in real-time. Thus, we cannot address more mechanistic questions such as whether recanalization was preceded by a change in vessel tone or a bout of neural activity. Future experiments imaging capillary diameter changes, blood flow velocity and/or neuronal calcium transients in awake behaving mice would be required to fully address these questions. Another caveat with our study is the use of microspheres to induce capillary obstructions. Although they do not recapitulate the components an embolus, microspheres offer many experimental benefits such as the precise timing of induction (which was critical in the present study) and the ability for high throughput testing of therapeutics. For example, the microsphere model previously identified Vascular Endothelial Growth Factor (VEGF) as a crucial factor in regulating capillary obstruction clearance (Reeson et al., 2018), which has now been successfully applied to improving capillary blood flow in a mouse model of Alzheimer’s disease (Ali et al., 2022). Of course, naturally occurring capillary obstructions arise from many different types of blocks or plugs, from cellular debris to a variety of circulating cells (Rapp et al., 2008; Santisakultarm et al., 2014; Cruz Hernández et al., 2019). Thus, a limitation of the microsphere model is the absence of biochemical interactions found in many obstructing cells, such as leukocytes with the vascular endothelium (Cook-Mills and Deem, 2005; Cerutti and Ridley, 2017). While NVC could modulate cell adhesion protein expression in endothelial cells or alter cell-cell interactions in the capillary lumen, it is possible that some obstructions (i.e., white blood cells) could be resistant to the effects of NVC. This may explain the surprising finding that leukocyte based capillary obstructions were not improved by exercise in an Alzheimer’s mouse model (Falkenhain et al., 2020). However, Alzheimer’s disease is also associated with abnormal cortical excitability and NVC (Park et al., 2020, p. 202; Li et al., 2021; Shabir et al., 2022), therefore exercise may not have sufficiently engaged NVC signaling pathways to overcome capillary stalling. Nonetheless, whether the present findings will have utility in clearing naturally occurring obstructions in aging or disease states where inflammatory factors are in play, remains to be determined. At the very least, we are encouraged by the fact that whisker stimulation in healthy adult mice can reduce capillary stalling events (Erdener et al., 2019).

There still remains significant uncertainty regarding the extent to which capillary obstructions impact cortical function. Despite the fact that spontaneous stalls or obstructions are relatively sparse at any given moment, we have shown that a single obstruction can perturb adjacent capillary flow for weeks (Reeson et al., 2018). Likewise others have shown that a relatively small number of obstructed capillaries can cause compound effects, leading to large reductions in overall blood flow (Cruz Hernández et al., 2019). Furthermore, our previous work has suggested that the accumulated loss of obstructed capillaries over a lifetime can significantly reduce cortical capillary density (Reeson et al., 2018). Interestingly, a recent paper from the Rochefort lab showed that under food deprivation, neurons in the mouse primary visual cortex downregulated AMPR expression to persevere energy use associated with action potentials, at the cost of introducing extra noise into neuronal encoding (Padamsey et al., 2022). This work suggest a frame work where impaired blood flow, despite being sub ischemic, leads to metabolic imbalances which in turn perturb neuronal encoding. Despite this evidence there remains many unanswered questions as to how, and when, these impairments in blood flow reach a critical mass, which will be crucial to properly evaluating the efficacy of any intervention.

There is no doubt that we are only beginning to scratch the surface of neuro-vascular interactions that can drive or magnify diseases of the central nervous system, like dementia (Iadecola, 2013). This creates a pressing need to understand how vascular changes in aging, such as reduced cerebral blood flow and capillary density, come about and what can be done to mollify these insidious processes. Increasing behavioral engagement through environmental enrichment may seem simplistic, however, it follows the sage idiom of “use it or lose it.” This seems particularly relevant to our elderly population who often encounter physical and social deprivation (Ong et al., 2016; Domènech-Abella et al., 2017). Our data indicate that simply increasing environmental richness is sufficient to improve capillary recanalization, and thus presumably, help maintain cerebral blood flow. Furthermore, the benefits of enrichment were realized in aged mice, proving that aging does not necessarily diminish one’s capacity to leverage behavioral engagement to restore capillary patency.

## Data Availability Statement

The raw data supporting the conclusions of this article will be made available by the authors, without undue reservation.

## Ethics Statement

The animal study was reviewed and approved by University of Victoria Animal Care Committee.

## Author Contributions

PR and CB conceptualized the study and wrote the manuscript. All authors collected and analyzed data, contributed to the article, and approved the submitted version.

## Conflict of Interest

The authors declare that the research was conducted in the absence of any commercial or financial relationships that could be construed as a potential conflict of interest.

## Publisher’s Note

All claims expressed in this article are solely those of the authors and do not necessarily represent those of their affiliated organizations, or those of the publisher, the editors and the reviewers. Any product that may be evaluated in this article, or claim that may be made by its manufacturer, is not guaranteed or endorsed by the publisher.
